# Helper-embedded satellites from an integrase clade that repeatedly targets prophage late genes

**DOI:** 10.1093/nargab/lqad036

**Published:** 2023-04-18

**Authors:** Dario Tommasini, Catherine M Mageeney, Kelly P Williams

**Affiliations:** Sandia National Laboratories, Systems Biology, 7011 East Avenue Livermore, CA, USA; Sandia National Laboratories, Systems Biology, 7011 East Avenue Livermore, CA, USA; Sandia National Laboratories, Systems Biology, 7011 East Avenue Livermore, CA, USA

## Abstract

Satellites such as phage-induced chromosomal islands (PICIs) are mobile genetic elements relying on helper phages for their mobilization, through trans-regulatory interactions. We discovered a PICI with a more intimate *cis-*regulatory configuration, integrated within a late gene of its helper prophage. This helper-embedded PICI (HE-PICI) configuration delays expression of the interrupted helper late gene until the satellite excises and provides passive helper-driven components to both HE-PICI replication and late transcription. Upon induction of a helper-satellite composite, precise excision of the entire composite was observed, followed by composite replication, then satellite excision. We mapped 491 additional HE-PICIs to one of 14 sites in cognates of phage lambda late genes. Associated integrases form a single phylogenetic clade with subclades respecting the 14 site groups, exhibiting repeated tropism for prophage late genes as new integration sites evolve. Four ordered zones in a general gram-negative PICI genome organization are: an integration zone encoding integrase and AlpA, a dynamic zone encoding members of the Bro-N network of domain-swapping DNA-interactive proteins and immunity repressor RNAs, a replication zone, and a dynamic late zone in which clusters as large as 17 consecutive helper prophage late genes have been captured. Helper-embedded satellites present new dimensions in satellite/helper relationships.

## INTRODUCTION

Bacteriophage satellites are small mobile genetic elements that require specific helper phages for their mobilization (1–5). A large group of satellites with shared gene content includes the classical satellite P4, the gram-negative and gram-positive lineages of the phage-induced chromosomal islands (PICIs), and PICI-like elements (PLEs) found in *Vibrio cholerae* (6–14). Another *Vibrio* satellite, TLCϕ, differs from the above by its use of a filamentous, rather than tailed, helper phage (15). Satellites exploit virion structure and assembly functions of the helper, at the expense of helper virion yields. They can adapt to their helpers, exhibiting genetic interactions that can be extensive and reciprocal. For example, P4 can derepress early genes and activate late genes of its helper P2, and respond in the same ways to P2-encoded regulators (1).

**Figure 1.**
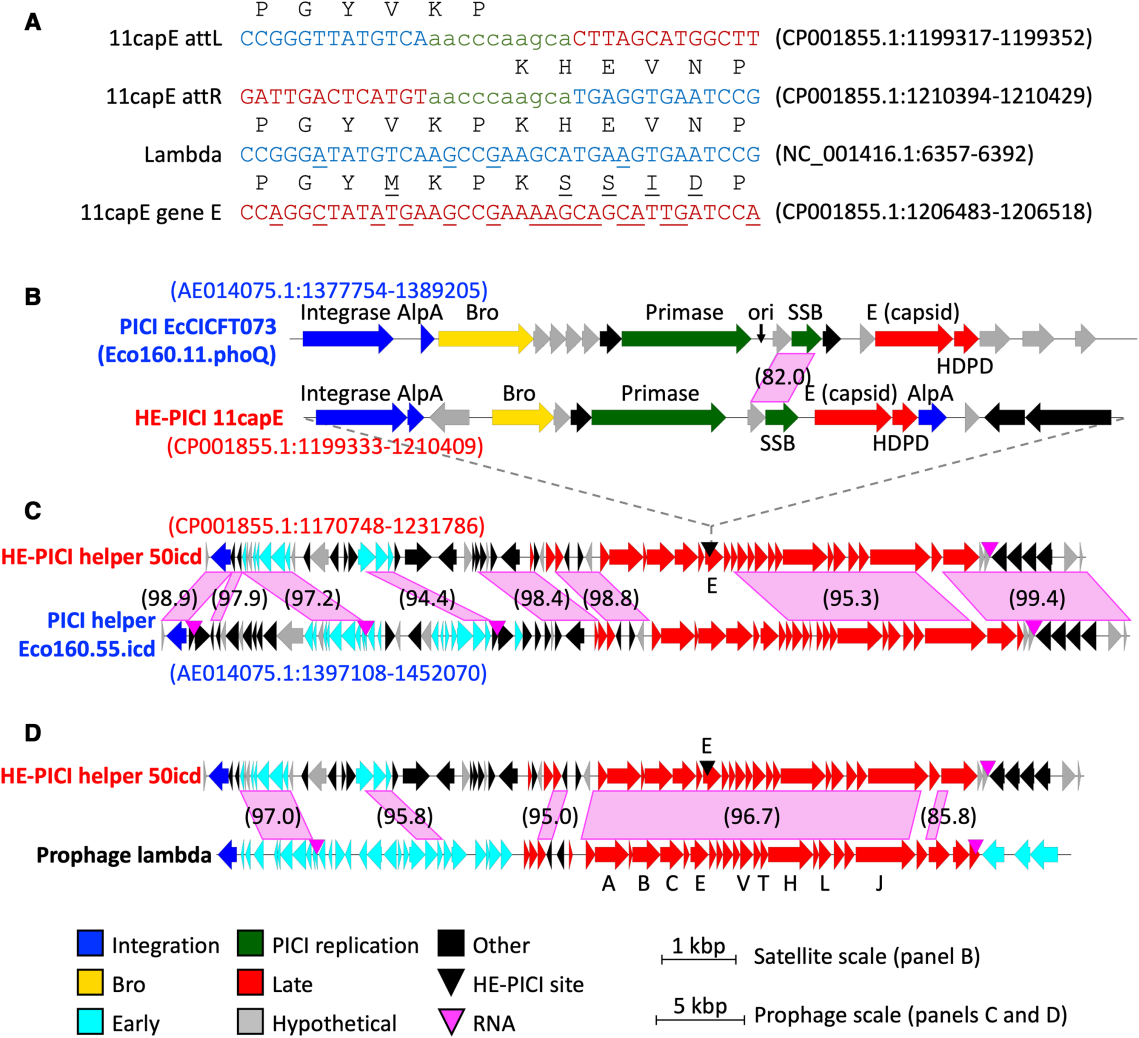
Satellite integrated within a prophage. (**A**) The integration site of 11capE in the capsid gene of prophage 50icd. The left and right *att* site sequences of satellite 11capE in the composite 61icd are shown with encoded capsid sequence above; prophage 50icd sequence in blue, 11capE sequence in red, and the *attL/attR* sequence identity block in green lower case. Corresponding segments are shown from the major capsid gene E of phage lambda and the homolog encoded within 11capE, with sequence differences from *attL/attR* underlined. (**B**) The 11capE HE-PICI component of 61icd shows similarities with model GN-PICI EcCICFT073 in size, gene content and order, and in a nucleotide sequence region; pink parallelograms show regions of nucleotide sequence similarity with percent identity in parentheses. The 50icd prophage component shows extensive similarity to the PICI helper prophage (**C**) and to phage lambda (**D**). Accession:coordinates given in parentheses for each sequence and island.

Although at least some satellites can optionally replicate in circular form like plasmids, they are known for integrating into the bacterial chromosome, and can thus be classified as genomic islands (GIs), as are their helper prophages. Each GI usually encodes an integrase that specifies its chromosomal integration (attachment) site, *attB*, and the corresponding site in the GI, *attP*. Integration converts *attB* and *attP* into the GI-flanking recombinant site pair *attL* and *attR*. The set of four *att* sites typically share a block of sequence identity of at least 7 bp, within which DNA strand crossover occurs during recombination. Our software TIGER (16) detects and maps GIs in bacterial chromosomes, with sufficient precision to delineate the *attL* and *attR* sites. TIGER revealed a nested pattern in one *Escherichia coli* genome, where a small element, 11capE, was integrated within a larger prophage, 50icd. Close similarity between 11capE and gram-negative (GN-)PICIs (6) and, separately, between 50icd and GN-PICI helpers (Figure 1) suggest a satellite/helper relationship between 11capE and 50icd.

Despite their recent recognition as a family, some interesting mechanistic aspects of the GN-PICIs have emerged (6,7). Three conserved genes are essential for induction and PICI transfer, *int* (integrase), *alpA* (a DNA-binding regulator) and *pri* (the replication primase); these, and most of their other genes, are unidirectional. AlpA activates its own promoter, and may (17) or may not (6) activate the upstream *int* promoter; relatives are also known as recombination directionality factors or excisionases (18). An origin of replication (*ori*) is found downstream of *pri*. GN-PICIs can carry phage late genes, most often a gene for the major capsid protein, which is present in 21 of 29 reference PICIs; however, such PICI capsid genes are not required for helper-assisted PICI transfer (6). GN-PICI genomes are small (10–20 kb), yet an *E. coli* model is packaged into longer, phage-length (∼45 kbp) DNA concatemers in the virion (6), unlike P4 and some other PICIs which are packaged in single-genome DNA lengths in smaller virion heads.

Here, we monitored induction of the 11capE/50icd helper/satellite, observing excision of the composite, followed by replication of the composite and excision of the satellite. Bioinformatic analyses revealed numerous relatives of the 11capE satellite among the Enterobacteraceae, each integrated within one of nine prophage late genes, and co-oriented with the late gene. This configuration, which we term helper-embedded PICI (HE-PICI), adds a new dimension to satellite/helper relationships.

## MATERIALS AND METHODS

### Bacterial strain

The 11capE HE-PICI-bearing strain, *E. coli* NRG857c (abbreviated here Eco567), was kindly supplied by Alfredo Torres (U. Texas Medical Branch).

### Phase 1 bioinformatic identification of HE-PICIs and other GIs

Our founding HE-PICI (11capE), its surrounding unit (61icd) and other prophages were identified and mapped in the chromosome (CP001855.1) of Eco567 using TIGER as previously described (16). Additional GIs were discovered as follows (summarized in Supplementary Table 1).

To examine relatives of 11capE, its integrase sequence was used as a TBLASTN v2.6.0+ (19) query with an *e*-value cutoff of 1e–20 against 165 778 proteobacterial genomes of reasonable quality (N50 > 10 000; < 300 contigs) downloaded from GenBank in July 2019, that were split into seven databases as follows: 58 711 *Salmonella*, 27 909 other Enterobacteriaceae, 2499 other Enterobacterales, 24 897 other Gammaproteobacteria, 30 634 *Campylobacter*, 1945 other Epsilonproteobacteria and 19 183 other Proteobacteria. The top five hits (highest bit scores) in each database were examined for phage gene surroundings, which were found only among the Enterobacterales. All the non-Enterobacterales integrase matches scored <400 bits; this cutoff was applied, leaving 5068 enterobacterial integrase genes (*int*s) for further examination. Regions of ∼22 kb (the *int*-matched segment with flanks of 1 kp upstream and 20 kb downstream) were taken, rejecting *int* regions containing an ambiguous base or truncated due to reaching a contig end, leaving 3490 *int* regions. During TIGER processing it became clear that non-HE PICIs integrated into the *fis* gene were an abundant artifact; jobs were cancelled for 230 yet-unprocessed regions containing a *fis* gene. TIGER (github.com/sandialabs/tiger) yielded 3177 satellite calls among the 3260 treated regions, although 14 of these required curation (splitting) of the annotation of an integration-generated gene fusion between *int* and the upstream prophage late gene fragment. For the 83 *int* regions where TIGER failed, we reapplied TIGER to the whole genome; this succeeded in mapping 32 more satellites which were often elongated beyond the initial ∼22-kb region. For eight additional cases, an HE-PICI was fully mapped manually by inspection of split late genes in the region; TIGER failure in these cases was explained by an insertion sequence very close to *attR* (one case) and by occurrence in the genus *Arsenophonus* which only had seven reference genomes in the database (five cases). This left 43 regions where at least one terminus of the satellite could not be mapped; for 34 of these, the putative target gene could be guessed based on the coding sequence and *attL* upstream of the integrase gene, but *attR* could not be identified, while nine remained fully unmapped, often associated with transposase genes near *int* that suggested chromosomal rearrangements. Three calls were recognized as two-PICI tandems that we split into their components.

For all genomes with a fully mapped HE-PICI, TIGER and Islander were run for the entire genome, delineating 208 HE-PICI-surrounding lambdoid helper prophages, and 24 *E. coli* HE-PICI-surrounding PICIs with the HE-PICI integrated into the PICI late gene site C:CLP_protease:110. For all these, orientation was assigned as that of the HE-PICI, and the hypothetical form with the HE-PICI excised was taken for further analysis. This yielded a total of 3452 new fully-mapped GIs, comprising 208 prophages, 2725 PICIs, and 519 HE-PICIs (Supplementary Table 1, Supplementary File 1). We added reference GIs to our study set—28 reference GN-PICIs (6), their one known helper (Eco160.55.icd), P4 and its helper P2, and lambda – for a total of 3484 study GIs. Reanalysis of reference PICIs by TIGER, Islander and inspection remapped 15 or more to tRNA genes (Supplementary File 1). To summarize taxonomy, all GIs studied in this Phase 1 survey are from 33 genera of the Enterobacteriaceae, except for reference PICIs from the Pasteurellaceae genera *Aggregatibacter, Necropsobacter and Pasteurella*.

### Gene content

The 3484 study GIs were annotated using the Tater pipeline (20), which supplements the Prokka (21) annotations with Pfam (22) HMM hits (http://hmmer.org), and carefully annotates integrase genes.

Initial clustering of proteins revealed that domain-swapping Bro network proteins were improperly aggregated, so they were treated specially. Primary hits to 10 Pfam HMMs of the Bro network were found among the GIs: ANT, AntA, Bro-N, KilA-N, ORF11CD3, ORF6N, P22_AR_C, P22_AR_N, Phage_ASH, and Phage_pRha. To account for possible new Bro domains, the flanking peptide sequences of these hits were used as BLASTP queries (evalue 0.00001) to find additional candidate Bro proteins. These proteins sequences were split into their hit and unhit segments; the segments were clustered using MCL (23) with an inflation parameter of 1.1), and aligned using MAFFT v7.453 (24) in ‘auto’ mode. The alignments were curated manually, including both breaking clusters into MAFFT -aligned subsets and splitting segments further based on closely related blocks (Supp. File 2). Each of the 144 groups of candidate Bro segments was designated with a ‘b’ followed by a serial number. To prevent spread into non-Bro proteins through occasional frameshifts, we rejected 24 rare Bro segments found more frequently outside than inside the original candidate Bro proteins. Since Prodigal occasionally may have miscalled ORFs, each segment was used as a TBLASTN query (cutoff 30 bits) against the GI genomes, identifying candidate Bro genes, each labeled according to its sequence of Bro segments.

Annotations in raw tater GFF outputs that shared a stop codon with a Bro protein were removed and Bro protein annotations were merged in. TBLASTN with AlpA queries showed that Prodigal had failed to call the AlpA reading frame in five satellites, each corrected. With the Bro proteins and the integrase family (Int) treated, the remaining 10334 unique proteins were treated by all vs. all BLASTP, with a cutoff of 30 bits, and clustered using MCL (23) with an inflation parameter of 1.4, producing 1395 families (682 singletons). A large family containing hits to Pfams Pox_D5, D5_N, DUF927, Prim-Pol or Prim_Zn_Ribbon was renamed ‘Primase’. Otherwise each family was named according to its most frequent Pfam hit, or if none, was considered hypothetical and named ‘H’ with a serial number (847 families). The family names of all encoded proteins were concatenated in genomic order, preceded with a ‘–’ symbol if oriented backwards, to produce 645 unique gene profiles, 169 for prophages and 476 for satellites.

The 26 families with either ‘tail’ or ‘holin’ in their names (all with late gene functions) were used as seeds to collect clusters of continuous co-oriented genes from the prophage profiles. Such clusters do not always perfectly separate early from late genes. For example, late genes lie downstream of and co-oriented with the lambda early gene Q, and an operon of two non-seed P2 late genes is transcribed oppositely to the other late genes. However this simple approach was mostly effective at separating known early and late gene families for both lambda and P2 (Supplementary Figure 1) except for low-abundance families in the situations mentioned above. An additional 32 families enriched in late clusters and associated with late phage functions were added to the seed and known late gene families, for a set of 93 high-confidence families categorized as Late, that account for 39.3% of all prophage profile genes. Other categories applied were Early (the known early gene families of lambda and P2 only), Integration (Int, Phage_AlpA and HTH_17 families), Replication (Primase, SSB and DUF5906 families), Bro (the Bro proteins), Hypothetical and Other.

Genome annotations and BLASTN comparisons were visualized according to gene content using EasyFig (25).

### *attP* sequence analysis

For the model GI for each of the 645 gene profiles (above), the *attL* and *attR* sequences were collected (Supplementary Files 3 and 4) and the *attL/attR* identity block (Supplementary File 1), were taken from TIGER output. In silico crossover between *attL* and *attR* within the shared identity block was then used to recontruct the hypothetical recombinant *attP* sequence of the excised GI. The *attP* sequences were aligned for HE-PICIs and separately for the *fis* PICIs, using MAFFT –auto, allowing discovery of the doubled motif.

### Phylogenetic analysis

From the 3527 study GIs, 3533 proteins were hit by the Phage_integrase Pfam HMM; four prophages lacked integrase genes and ten PICIs contained secondary integrase genes outside the usual integration module. These were combined with the 24 integrase sequence fragments used to seed the Pfam family Phage_integrase (22). Sequences were aligned using ‘mafft –auto’. RAxML v 8.2.6 (26) was used to prepare the best maximum-likelihood tree from 20 started with randomized stepwise addition parsimony trees (raxmlHPC-PTHREADS -m PROTGAMMALG -p 12345 -# 20), and support values were taken from 250 bootstrap replicates (raxmlHPC-PTHREADS -m PROTGAMMALG -p 12345 -b 12345 -T 128 -# autoFC).

### Prophage induction

Three replicates were performed simultaneously, each started from a different colony of Eco567 grown as an overnight culture, which was diluted 100-fold and grown in LB to mid log phase (OD_600_ = 0.4–0.5). Mitomycin C was added to 1 μg/ml and 1-ml samples were taken at 0, 0.5, 1, 2 and 3 hr after treatment. Samples were centrifuged at 5000 × g for 2 min. The supernatant was removed and passed through a 0.22 μm filter. Genomic DNA was isolated from the pellet with the DNeasy Blood & Tissue Kit (Qiagen). Genomic DNA yields were quantitated using Qubit Flex Fluorometer (Thermofisher).

### Quantitative real-time PCR (qPCR)

PCR products (Supp. Figure 2) were prepared using QIAquick PCR Purification Kit (Qiagen) and quantitated using Qubit Flex Fluorometer (Thermofisher), as standards for each of the eight *att* site forms of interest (*attB, attP, attL*, and *attR* for both 61icd and 11capE), using the primers listed in Supplementary Table 2. Genomic DNA samples of 40–60 ng were prepared, along with a hundred-fold dilution series (100 pg, 1.0 pg, 10 fg and 100 ag) of standard curve samples. PCR reactions were performed in PowerUp SYBR Green Master Mix (Applied Biosystems) with each primer at 500 nM using a three-step amplification protocol (58ºC annealing) in a CFX96 Touch Real-Time PCR Detection System (Bio-Rad).

The slope and intercept of each molar standard curve (all *r*^2^ values were above 0.98) was taken, and the following formula used to convert from Ct (cycle threshold) value to copy number in the genomic DNA sample: copies = 10^(Ct-intercept)/slope^. All reaction efficiencies were above 88%. The total copy number of *icd* regions was taken as: regions = B + (L + R)/2, where B, L and R are the experimental *icd attB, attL*, and *attR* counts, respectively. Finally, *icd attB* and *attP* counts were reported after normalizing to *icd* region counts.

### Quantitation of *att* sites through deep sequencing

Deep sequencing was employed as a second method of measuring *att* sites, through counting of reads from each *att* site type (27). Sequencing libraries were prepared from the genomic DNA samples of one of the induction experiment replicates using the Nextera DNA Flex library prep kit with the Nextera DNA CD Index kit (Illumina). Libraries were quantitated using the Qubit high-sensitivity DNA assay kit (Thermofisher Scientific) and pooled in equal quantity to form a multiplexed final library, requantitating with Qubit and confirming quality with the Agilent bioanalyzer using high sensitivity DNA chip (Agilent Biotechnology). The final library was sequenced using Illumina technology on a NextSeq 500 sequencer with a high-output 150-bp single-end read kit. Sequencing read sets have been deposited in the Sequence Read Archive (PRJNA928767).

Sequencing reads were quality filtered using BBDuk (http://jgi.doe.gov/data-and-tools/bb-tools/). The quality filtered reads were analyzed with Juxtaposer (27) to identify mobile genetic elements and AttCt (27) software to determine *attL, attR, attB*, and *attP* read counts for each GI. Bioinformatic probes used by AttCt for each predicted genomic island are listed in Supp. Table 3. The *attB* and *attP* read counts were normalized to *icd* region (or *torS* region) counts as described above for qPCR.

### Phase 2 search for *fis* GIs and their analysis

In recent work to be reported elsewhere, we applied TIGER to all of the 165 778 proteobacterial genomes. The *fis* gene was called in these genomes by PROKKA and this annotation is transferred to the name of any GI integrated into the gene. Among these TIGER calls, 3560 were found to be labeled *fis*. To resolve any possible remaining tandems, each call was searched internally for the *fis att* consensus identity block and its known variants among the Phase 1 GI set. Six cases of internal *att* sites were found. One (Cfr95.9.fis) had two copies of *attR* surrounded by a block of Ns; this assembly artifact was easily corrected by trimming to the internal *attR*. The other five cases were resolved into tandems: three from *Hafnia* strains (Hal39.20.fis, Hal10.20.fis, Hal26.20.fis) all had the same split into a 17 and a 3 kb component. Another (Sne13.17.fis) was split into a tandem of a 15 and a 2 kb component. The last case (Ecl37.33.fis) was split into two 17 kb components, although we suspect that one is an assembly artifact caused by partial activity of the PICI, because it is identical to the other except for a 3297-bp block of Ns. This left 3565 *fis* GIs, 2642 of which were present in our original set. The 923 new *fis* GIs broadened coverage beyond the Enterobacteriaceae, to include Aeromonadaceae, Alteromonadaceae, Shewanellaceae, and Vibrionaceae.

To examine whether any of these new *fis* elements might be prophages we collected 422, 92 and 83 unique proteins respectively hit by the signature Pfams Phage_AlpA (PICI signature), or Phage_min_tail or Phage_tail_L (prophage signatures), in any of our GIs using hmmsearch against the three Pfam release 35.0 HMMs with ‘trusted cutoffs’. These were used as TBLASTN queries (evalue = 0.00001, max_target_seqs = 500 000) against model GIs and the new *fis* GIs.

## RESULTS

### Nested pair of genomic islands

GIs become integrated into the bacterial chromosome through the action of a GI-encoded integrase which catalyzes recombination between the *attP* site in the GI and the cognate *attB* site in the chromosome, yielding the integrated GI flanked by recombinant *attL* and *attR* sites. Our comparative computer program TIGER (16) employs a ping-pong BLAST method that precisely maps GIs in a query genome, by finding closely related reference genomes in which the *attB* site is intact (uninterrupted by any GIs). TIGER outputs coordinates for the GI and its *attL* and *attB* sequences, from which the *attP* can be reconstructed. In a search for GIs that encode a homolog of the same chromosomal gene into which they integrate (16), we found in *E. coli* NRG857c (Eco567) an 11-kbp GI, termed Eco567.11capE (Supplementary File 1). Our GI nomenclature combines an abbreviation and serial number for the strain with the size and the gene target of the island. 11capE is integrated within (and disrupting) a phage capsid gene, yet bears its own capsid gene. 11capE was nested within another GI call, 61icd, that TIGER identified as a prophage; its integration site in the *icd* gene is a well-known prophage integration site in *E. coli* (28). We hypothesized that 61icd is a two-part composite, with the 11capE component integrated into the capsid gene of a putative 50-kbp prophage that we term 50icd. Sequence comparison of the left and right attachment sites (*attL* and *attR*) of 11capE (Figure 1A) reveals a 10-bp sequence identity block where integration occurred. The 50icd capsid gene is a very close relative of the major capsid gene E of phage lambda, and the capsid gene within 11capE is from the same family, but extensive sequence differences in the region corresponding to the attachment site would likely prevent 11capE self-integration.

11capE shares the size and gene organization of the recently discovered gram-negative PICIs, and some sequence similarity, as shown in Figure 1B for the emerging model PICI (6,7), EcCICFT073. The helper prophages of 11capE and EcCICFT073 likewise show extensive similarity to each other (in this case sharing the same chromosomal integration site in the *icd* gene), and to phage lambda (Figure 1C, D). Whereas other PICIs are integrated into chromosomal genes apart from their helpers, allowing interaction only *in trans*, 11capE is phage-embedded. Integration within its helper prophage allows a unique *cis-*regulatory relationship between satellite and helper that we discuss below. Clearly, expression of the 50icd capsid gene cannot occur until 11capE excises. Furthermore, both late transcription and replication of the helper should also propagate passively to the integrated satellite. We term this new nested configuration a helper-embedded PICI (HE-PICI).

### Induction: excision and replication of the composite, and excision of the satellite

**Figure 2.**
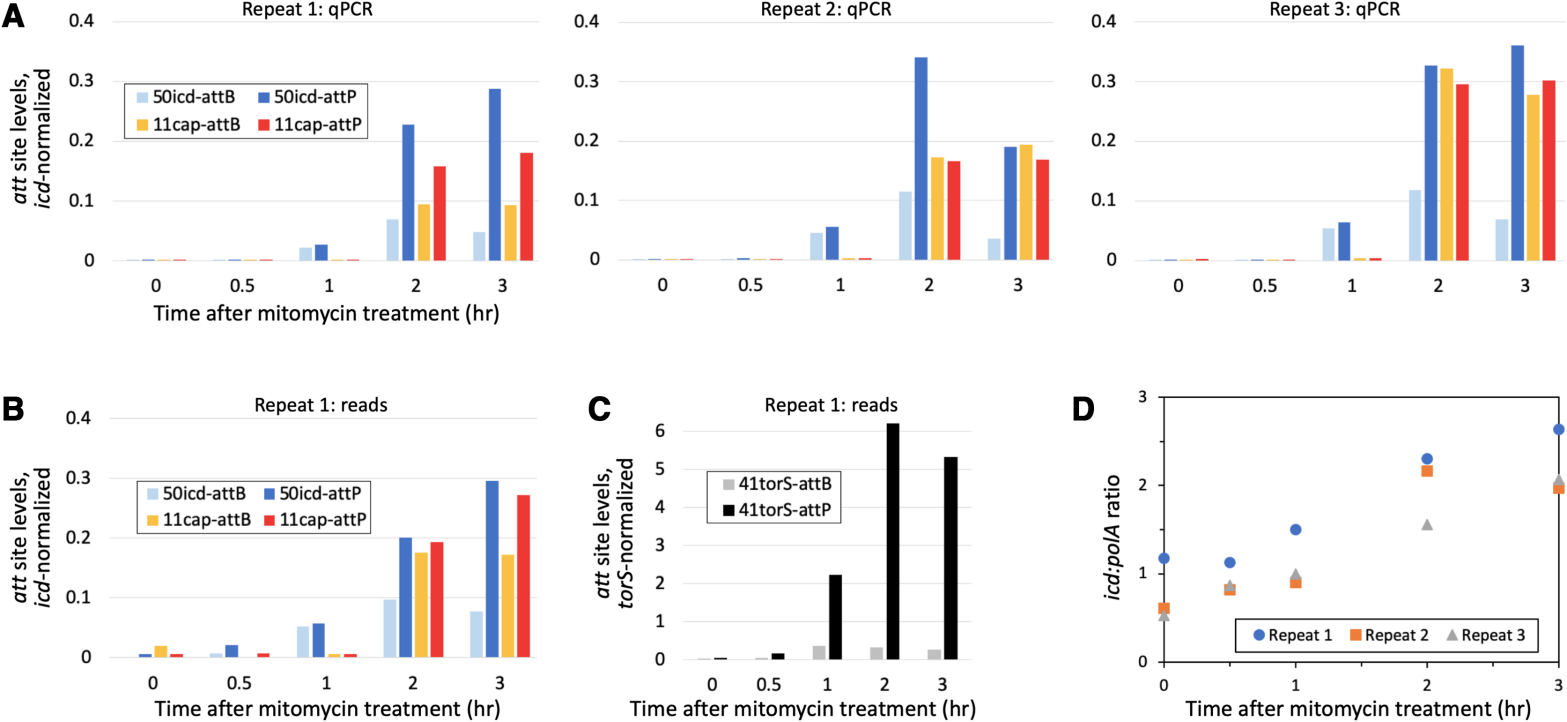
Monitoring recombinant attachment site (*attB* and *attP*) levels after induction. (**A**) Three independent replicates of a mitomycin prophage induction experiment were monitored by qPCR. Counts of *att* sites are normalized to those of the *icd* gene as described in Materials and Methods. (**B**) Replicate 1 was subjected to deep sequencing and read counts were taken for each *att* site, normalizing as in (A). (**C**) Deep sequencing *att* count data for 41torS, normalized to the *torS* gene. Note the 15-fold y-axis increase relative to (B). (**D**) In situ amplification of *icd* upon induction, normalized to the distant housekeeping gene *polA*, measured by qPCR.

Induction is a key stage in the life cycle of a temperate phage, a stage where a satellite and helper may interact most (11). The HE-PICI:helper lysogen Eco567 was treated with a standard prophage inducer, the DNA-damaging agent mitomycin C, and the status of the attachment sites of 61icd and 11capE was monitored over time, using two quantitative methods, qPCR and read counting from deep sequencing (27) (Figure 2A, B). Of special interest are the products of excision, *attB* (the chromosomal scar) and *attP* (the circularization junction of the mobile element), while the *attL* and *attR* pairs of the unexcised lysogen are used in normalization. Reads from deep sequencing confirm precise excision of both 11capE and 50icd, producing their TIGER-predicted *attB* and *attP* sites, and likewise for another predicted Eco567 prophage, 41torS.

First, we measured *icd attB*, representing derepression and excision of 61icd, as well as the *attL* and *attR* that indicate non-excision, to compute the *icd*-normalized *attB* levels for 50icd, a metric for the extent of induction. The *icd attB* is detected at 1 h post-treatment, at levels corresponding to induction in 2–5% of the *icd* genes, rising to a maximum of only 7–11% at 2 h, and dropping back to 3–7% at 3 h. We interpret this late drop of normalized *attB* as overgrowth by the much larger number of healthier uninduced cells. The separate prophage 41torS is more strongly induced, in up to 35% of *torS* genes (Figure 2C). No reads corresponding to *attB* or *attP*, indicating excision, were found for a third TIGER-predicted prophage, 45proP.

Extent of post-excision 61icd replication was monitored. Levels of *icd attB* and *attP* would be nearly equal if there were no replication following excision. Instead we observed an excess of *attP* relative to *attB*, evidence that the excised helper/composite replicated faster than the chromosome. Ratios of *icd attP:attB* were only 1.1–1.2 by 1 h, but rose to 2.1–3.3 by 2 h and to 3.8–6.0 by 3 h, indicating post-excision replication of the helper/composite. By contrast, excised 41torS replicated even faster, reaching an *attP:attB* ratio of 20. The other key excision event, of 11capE, was not detected until the later timepoint of 2 h, when substantial replication of the 61icd composite had occurred. The ratio of the 11capE *attB* to the *icd attP*, a measure of the extent of excision of 11capE, was 42–98% at 2 and 3 h. Experimental repeat 2 showed a delay in excision relative to replication, with an excision yield of ∼50% at 2 h and ∼100% at 3 h. 11capE *attP* and *attB* levels were nearly equal in two of the three experimental repeats. This suggests either that neither of the separated 11capE and 50icd components replicated further after satellite excision, or that they both replicated at equal rates. In the other repeat, the *attP:attB* ratio was at 1.6–1.9, suggesting moderate post-excision replication by 11capE.

In addition to replication of 61icd after excision, there was amplification of unexcised 61icd, evidenced in a steady increase (2.2–3.9 fold over 3 h) in the ratio of the *icd* gene to the distant *polA* gene (Figure 2D). This comports with *in situ* pre-excision replication observed for P2 and other temperate phages (29–32).

Our attempt to isolate 50icd or 11capE phage particles failed. We prepared lysates from induced Eco567 cultures, but could not detect plaque-forming units either on lawns of *E. coli* MG1655 or on lawns of Eco567 from which we had precisely deleted 61icd (data not shown).

### Additional HE-PICIs

**Figure 3.**
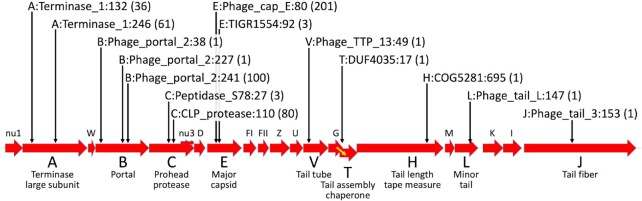
HE-PICI integration sites in prophage late genes. A 19-kb region of phage lambda late genes is shown. Each HE-PICI was found in a homolog or functional cognate of one of these late genes, oriented such that the key HE-PICI genes (integrase, AlpA, and primase) have the same (rightward) orientation as the late gene. The labels for the 14 detected HE-PICI sites have three parts: the lambda gene cognate, the protein family sequence profile that covers the integration site, and the position of the site within that profile; the number of HE-PICIs found at the site is in parentheses. The T gene has no start codon; it is only expressed upon a translational frameshift (yellow arrow) from the overlapping G gene.

We searched for more examples of 11capE by querying genome databases with its integrase sequence (Supplementary Figure 3, Supplementary Table 1). As expected we found more instances, totaling 201, of similar elements in the same site in homologs of the 50icd capsid E gene. Surprisingly, the related integrases also led to 290 instances of HE-PICIs in other phage late gene sites, totaling 14 sites in nine late genes (Figure 3). These 14 site types are standardized using protein profiles of the late proteins, named according to their lambda cognate gene, the protein profile encompassing their attachment site, and the location of the central codon in the attachment site within that profile. For example, 11capE is inserted in the 80th codon of the Pfam profile Phage_cap_E, in a cognate of phage lambda gene E, so we term this site E:Phage_cap_E:80. A fifteenth HE-PICI site (B:Phage_portal_2:21) was putatively identified, based on late gene coding sequence upstream of the *int* gene; its *attR* could not be located. We can describe this effort as a systematic survey, not for all HE-PICIs, but for HE-PICIs with close relatives of the 11capE integrase. It is very likely that there are more HE-PICIs in genomic databases; several of those found here were close to our integrase match cutoff score (Supplementary Figure 3).

For 209 HE-PICIs, the surrounding helper prophage was mapped; in one case the same helper prophage (Ana29.79.guaB) contained two HE-PICIs at separate late gene sites (A:Terminase_1:245 and V:Phage_TTP_13:49). For 24 additional HE-PICIs (all using the C:CLP_protease:110 site), the target late gene was not within a helper prophage, but instead mapped within a PICI itself integrated into a tRNA-Ile2 gene, forming a composite of only 20.6-kb. Other C:CLP_protease:110 HE-PICIs are in prophages, so the integration into PICIs may be accidental, but could nonetheless reveal interesting biology where the HE-PICI essentially has two helpers.

Of satellites mapped to chromosomal sites, 28 were related to a HE-PICI group, based on integrase phylogeny (see below) and confirmed by alignment of the *attL* and *attR* sequences (Supplementary Files 3 and 4). We interpret these as the result of off-target integration events by HE-PICI integrases, much like lambda and other temperate phages can integrate into secondary chromosomal *attB* sites if the normal *attB* is unavailable (33). Further proof of relatedness within off-target and on-target groups comes from island sequence comparisons; off-target Kpn6881.11.C_nth shares 99.97% sequence identity with on-target Kpn4002.11.C_CLP_protease_110 over their entire lengths.

The remaining chromosomal satellites were found at one of two sites, 2642 in the *fis* gene and 49 in the *hpt* gene, representing two new subfamilies of non-HE PICIs. Their integrases matched to 11capE just above the cutoff. A tandem pair of PICIs was noted at the *fis* gene in two genomes (Supplementary File 1).

Summarizing the categories of the 3452 mobile genetic elements newly mapped in this Phase 1 survey, we found 491 HE-PICIs, 28 off-target HE-PICIs, 2701 regular PICIs (at *fis* or *hpt* loci), 208 helper prophages surrounding HE-PICIs and 24 PICIs surrounding HE-PICIs. For each of these elements and for several reference elements from the literature, Supplementary File 1 reports the identity sequence shared by *attL* and *attR*, discovered by TIGER and precisely mapping the element, as well as accession:coordinates, helper/satellite relationships, and other information.

### Integrase phylogeny

**Figure 4.**
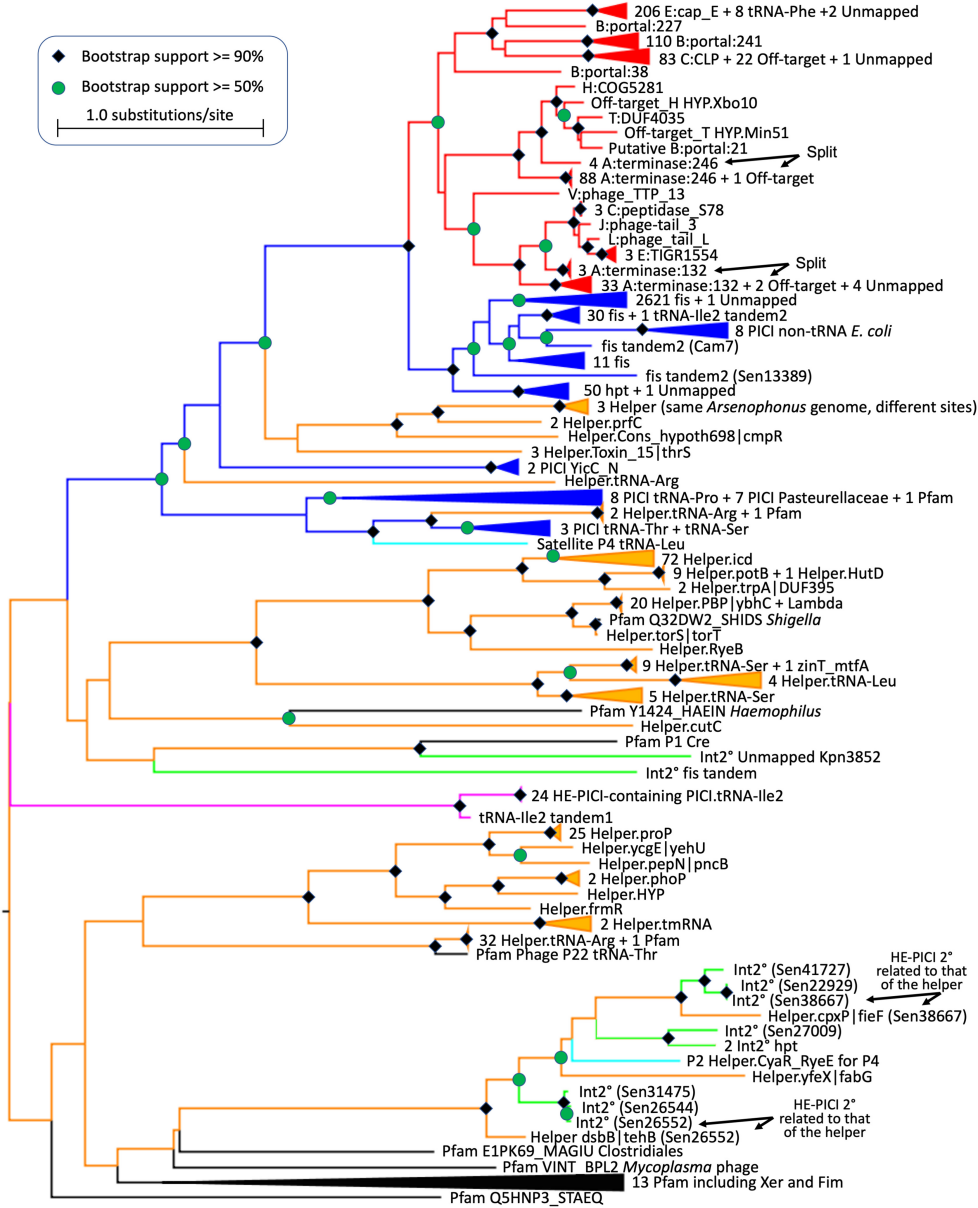
Integrase phylogeny. The primary integrase of each HE-PICI (red), reference or newly found GN-PICI (blue), helper prophage (orange) or surrounding PICI (magenta), unmapped elements, the secondary integrases encoded by some of these satellites (Int2, green), and reference Pfam seed integrases (black) were aligned to build a maximum-likelihood tree. Each putatively-mapped *attB* was confirmed by the tree and the clades containing the nine fully unmapped elements are indicated. For each helper clade, the *attB* site specificity is indicated.

All the new HE-PICIs, PICIs and prophages had a primary tyrosine integrase gene near a terminus of the element, and some contained secondary internal *int* genes. A phylogenetic tree was prepared for all these integrases, together with the integrases of the 48 unmapped satellites and several reference integrases (Figure 4). The observed unifying principle for integrase clades is the use of the same *attB* site. Perhaps unsurprisingly (since HE-PICIs were discovered based on integrase similarity), the primary HE-PICI integrases form a distinct clade. The HE-PICI clade constitutes a branch within a larger clade of mostly PICIs and satellite P4. Some helper integrases also fall in this PICI group, but most helper integrases are from a larger subtending group with integrases from phages lambda and P22. Helper prophages use a variety of chromosomal *attB* sites, and HE-PICI types can be mixed within helper clades using the same *attB*. The integrase tree confirms all 34 putative *attB* sites for the cases where *attR* could not be identified, and suggests origins for the nine fully unmapped cases. The integrase of the C:CLP_protease:110 HE-PICIs appears the most promiscuous, accounting for 24 of the 28 off-target events. Allowing for these off-targets, each of the 14 site-groups of HE-PICIs were coherent (monophyletic) by integrase phylogeny, except for the two slight splittings indicated. The 14 site-groups were further supported by *attL* and *attR* sequence alignments (Supplementary Files 3, 4). The integrase for the HE-PICI at the putative fifteenth site, B:Phage_portal_2:21, formed an isolated branch distant from those of the other three B:Phage_portal_2 sites. The secondary integrase encoded in nine HE-PICIs were found outside of the PICI region of the tree; in two cases (marked by arrows), a secondary HE-PICI integrase gene was found to be closely related to that of the helper prophage for the HE-PICI.

### Attachment sites

Tyrosine integrases (the only type used by the GIs studied here) tend to enforce sequence identity between *attP* and *attB* at the typically 7-bp segment where strand exchange occurs (34). This core of the *att* site identity block can extend substantially further (up to ∼250 bp), when an integrase specifies an internal site well within the target chromosomal gene, as occurs at *icd* and many tRNA genes (35). In these cases, the *attP* site carries a replacement sequence such that the target gene is still functional after integration. TIGER identifies such extended identity blocks by comparing *attL* and *attR* from the query genome to the uninterrupted *attB* of a reference genome, but cannot determine the crossover core within it. Subsequent comparison of diverse sequence instances helped localize the core in the larger HE-PICI groups and the *fis* and *hpt* PICIs.

As usual, many of the new prophage integrases target tRNA genes, and examination of reference and new PICIs revealed the same for many of them (Supp. File 1). Alignment of the tRNA fragments in the *attR*s (Supplementary File 4) shows the phenomenon of DNA damage (35) for Pro, Thr, selenocysteine and some Ser and Ile2 tRNA genes, i.e. deletions of variable length that start at a specific site in the acceptor stem (position 99 in Supplementary File 4). At least among our study GI set, all GIs inducing damage in tRNA gene fragments are PICIs, while none of the many prophages that target tRNAs damage the fragments.

The *attP* site for each GI was reconstructed, and a motif was found in HE-PICIs, which can be written as tgTGTATTGCantgTGTATTGCa, where lower case bases are less conserved (Supplementary Figure 4). This doubled (tandem occurrence of the TGTATTGC core) motif was not found among other PICIs, including the *fis* PICI *attP*s, whose integrases are close relatives to those of the HE-PICIs. The motif is found in two copies in each HE-PICI, precisely positioned relative to the presumed crossover (strand-exchange) region of the *attP*. One copy of the doubled motif lies 81 ± 2 bp upstream of the *attP* center and a co-oriented second copy lies 132 ± 2 bp downstream of the center. The four half-sites may be bound by the arm domains of the integrase homotetramer, or could be bound by the HE-PICI AlpA or some unknown DNA binding protein; the motif does not resemble the IHF binding site at *attP*s for other integrase clades.

### Gene content

We have generated a large database of novel PICIs and used them to make some generalizations about PICI gene organization. Proteins of the HE-PICIs, PICIs and helpers discovered here, together with reference GIs (3484 total) were clustered into families based on similarity scores. This provided a standardized gene profile to each fully-mapped GI, reducing the GIs to 645 unique genotypes—220 for HE-PICIs and their off-targets, 256 for the PICIs, one for P4, and 169 for the prophages (Supplementary Figure 5). This reduction acts to correct redundancy due to over-representation in the genome databases of the genera *Escherichia, Salmonella* and *Klebsiella*.

**Figure 5.**
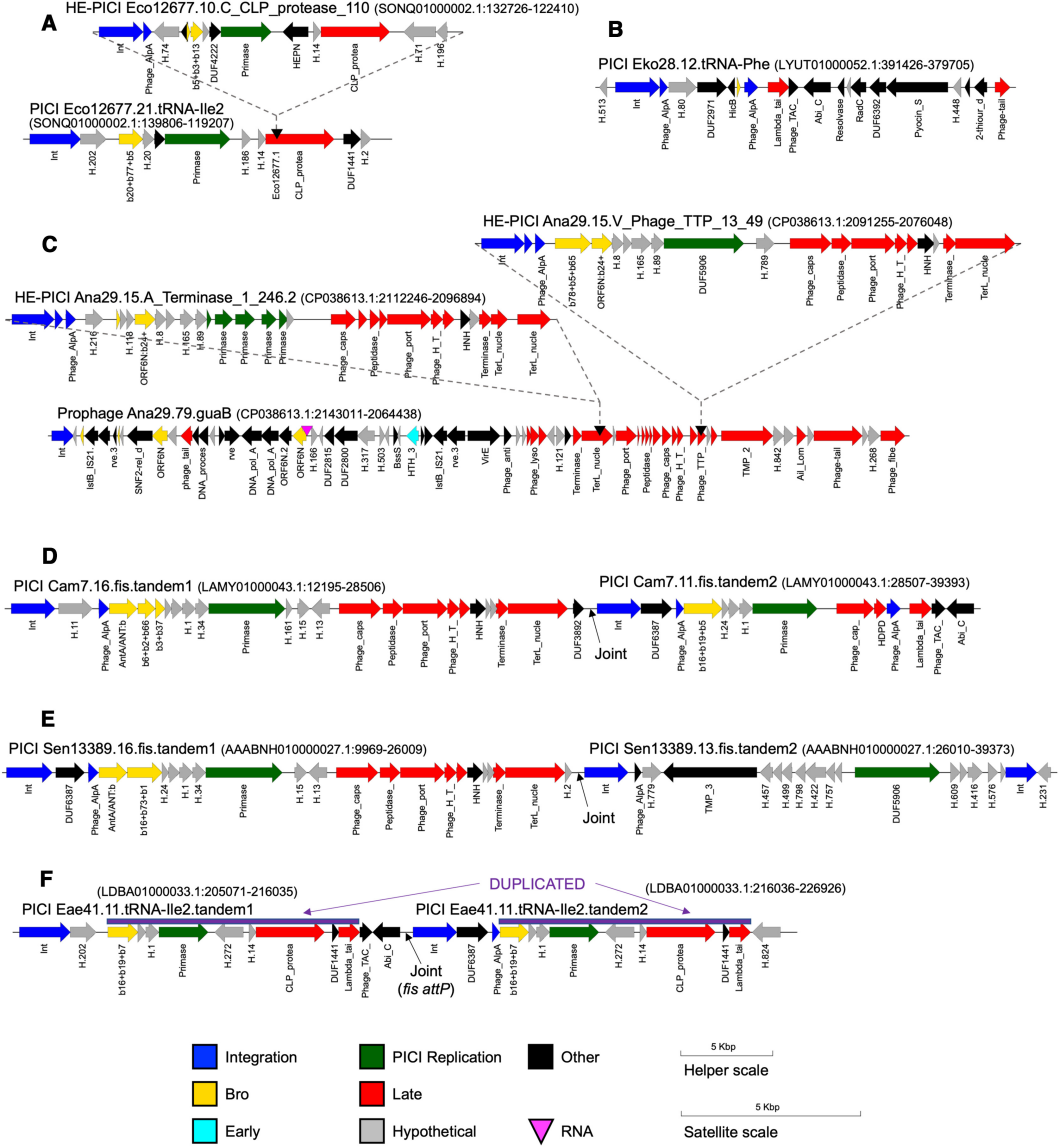
Variant satellite configurations. (**A**) HE-PICI in a PICI. (**B**) PICI in a tRNA gene, though using the same integrase subclade that targets the cap_E late gene. (**C**) Helper with two separate HE-PICIs. (**D** and **E**) Simple tandems in *fis*. (**F**) Complex tandem between a tRNA-Ile2 PICI and a *fis* PICI, where the two share a long duplicated region. Accession:coordinates for each satellite are in parentheses.

We first note some peculiar satellites: (i) One group is integrated in tRNA-Phe genes, despite having an integrase extremely similar to that specifying the E:Phage_cap_E site; these lack a Primase and uniquely encode a Resolvase (Figure 5B). (ii) The PICIs invaded by HE-PICIs shared a single genotype, bearing a CLP_protease gene containing the HE-PICI *attB* (Figure 5A). These PICIs are integrated into an tRNA-Ile2 gene, with integrases that are phylogenetically quite distinctive (pink lines in Figure 4), and also lack an *alpA* gene. (iii) A tandem satellite pair was found within a tRNA-Ile2 gene, with one integrase similar to those above that specify tRNA-Ile2 (Figure 5F). Normally the three *att* sites defining a tandem pair have similarities, but in this case, the terminal *att* sites are adapted for tRNA-Ile2 and not related to the internal *att* site which is a *fis* PICI *attP*. Thus, it is difficult to envision how the two units would join by integrase activity. Perhaps a circular *fis* PICI entered the tRNA-Ile2 PICI by homologous recombination within the long segment that is duplicated in the tandem.

We observed four ordered zones among HE-PICIs and PICIs in the upstream to downstream direction: Integration, Bro, Replication, and Late. In the Integration category, with Integrase proteins, we include HTH_17 and the multifunctional Phage_AlpA, known as excisionases controlling the directionality of integration (18). The Bro zone is dominated by domain-swapping proteins of the Bro network (36). Their structural similarity to homing endonucleases has been noted, suggesting possibilities for DNA recombination activity. A member encoded by *Vibrio* satellite PLE has been shown to act as a repressor (37). The dynamism of these Bro network genes among the satellites required a specialized annotation approach (Methods) that provides further evidence of domain swapping. Also encoded within some Bro zones are immunity RNAs; In P4, the RNA repressor CI is encoded within a Bro network gene and controls early transcriptional termination by binding to a nearby RNA site, seqA (38). Two Rfam profiles (C4 and isrK) can detect CI and seqA relatives (39), and these hit several of our GN-PICIs and HE-PICIs. The Primase, SSB and DUF5906 proteins, whose genes colocalize along with mapped origin of replication sequences (6), were placed in the Replication category.

The downstream Late zone, like the Bro zone, is another dynamic region of PICIs, containing diverse prophage late genes; we propose that the late genes in PICIs can be captured in clusters from their own prophage helpers. Late genes were identified by co-oriented gene clusters from our own prophage set (Supplementary Figure 1) and from two model *Escherichia* prophages. Our late gene categorization system was conservative and did not find late genes among the most divergent satellites, the reference PICIs of the Pasteurellaceae. This may reflect bias by our system toward annotation of late genes that operate in *Escherichia*; indeed capsid genes have been identified by others in most of these Pasteurellaceae PICIs (6), in our Late zone. A small number of PICIs are elongated; 11 profiles have PICIs longer than 22 kb (Supplementary Figure 5A, B, purple diamonds). The zone that is elongated in each of these is the Late zone, such that they contain more late genes than usual, although this is not shown well for the long PICIs from *Cedecea* (Cnf1.45.fis) and *Serratia* (Squ6.91.Cons_hypoth698|cmpR), genera whose annotation may not be served well by our conservative *Escherichia*-based system.

The elongated PICI Late zones suggest that PICIs may capture late genes from prophages. We found specific instances corroborating this idea. We found seven unique clusters of five or more genes shared between a PICI and a prophage; these were all in PICI Late zones (Supplementary Figure 6). In some cases these gene cluster matches also show similarity at the nucleotide sequence level (pink parallelograms). The longest PICI/prophage BLAST match detected was 7243 bp, between Sen29644.42.fis and Cfr111.52.RyeB, although with an average nucleotide identity of 87.5%. We do not observe perfect identity between any of the matching segments, and thus do not propose direct source/recipient relationships, but do propose that these matching prophages are relatives of the direct sources of PICI late gene clusters. The highest identity for long hits was found in two separate segments of HE-PICI Eco6102.18.E_Phage_cap_E_80 that are shared with the prophage Eco15043.56.R. The order of these two segments in the PICI is reversed relative to the prophage; interestingly, the two segments in the prophage abut at the Phage_cap_E_80 site of HE-PICI integration (downward black arrow), suggesting integrases may participate in capture. Other long BLAST hits between PICIs and prophages were insertion sequences (not shown), revealing little about PICI biology. Although at first glance the PICIs with elongated late zones may resemble phages, the conservation of the gene content of the first three zones (Integration, Bro and Replication), the conservation of their *attP* sequences (Supp. Files 3 and 4), and the conservation of their integrase sequences within the corresponding PICI subclades (Figure 4), distinguishes them as PICIs.

Bro network protein genes, such as P4 *eta*, can be the scene of multiple regulatory activites in satellites (40). The transcript from a constitutive promoter internal to *eta* is quickly terminated due to RNA interactions at the CI and seqA sites; processing of this transcript produces the stable immunity RNA CI. Homologs of CI and seqA were found in several of the HE-PICI/PICI Bro genes, suggesting that their Bro zones provide immunity functions. A truncated form of Eta (Kil, which functions in cell killing) can be translated upon occasional terminator read-through. Full-length Eta cannot be translated until an upstream late promoter is activated; *eta* is translationally coupled to the upstream *alpA* homolog. Eta translation prevents termination, so that the late promoter reads through the full *eta* gene and downstream genes.

Bro network proteins often have gene regulatory functions; they can mix domains related to nucleases with other domains that have a distinct DNA-binding fold (36). One example, CapR of the *Vibrio* satellite PLE. represses the capsid gene of its helper phage by binding its promoter −35 region (37). CapR fits into the Bro network by its similarity to domains in proteins with a Bro network nuclease domain. A protein (MJY48377.1) of the HE-PICI Eco11232.16.A_Terminase_1_246 contains the HNH_3 nuclease domain suggestive of a Bro gene mobilization mechanism; this domain is linked to the AP2 domain and the Bro segments termed here b60, b155, b3, and b22.

The four-zone gene organization (Integration, Bro, Replication, Late) unifies HE-PICIs with the other GN-PICIs (Supplementary Figure 5A, B). This organization furthermore distinguishes these PICIs from prophages (Supplementary Figure 5C), which display an Early/Late organization. Prophages do have an Integration module, but lacking AlpA and with the *int* gene aiming outward rather than inward.

### Probing late gene capture by additional analysis of *fis* GIs

We suggested above that the elongated PICIs result from internal capture of late gene clusters from prophages, and presented particular examples of such gene clusters derived from our own set of 169 model prophages (Supplementary Fig. 6). A possible objection to this interpretation is that the elongated PICIs are either unresolved adjacent PICI/prophage neighbors or PICI/prophage fusions that resulted from deletion of intervening DNA from between an original PICI/prophage neighboring pair. For deciding between the capture hypothesis and the adjacency/fusion hypotheses, it is simplest to focus on the chromosomal *fis* gene integration site, rather than the potentially more confusing HE-PICIs which are already known to integrate into prophages. This focus is especially valid because many more *fis* PICIs than HE-PICIs are available, and these include nine of our 11 elongated PICIs (Supplementary Figure 5A, B, purple diamonds).

First, we note that since we submitted our manuscript, a study has appeared showing gain of late genes within PICIs (41). Second, we repeat that the boundaries found by TIGER are extremely reliable (that is, precisely mapped). We find very strong sequence conservation of the identity blocks found within *attL* and *attR* at every *fis* PICI (Supplementary Figure 3C, Supplementary Files 3 and 4). The consensus is TACGGCATGAACTAA, corresponding to the terminus of the *fis* gene (the *fis* stop codon is underlined) which is typically followed by a rho-independent transcription terminator into which the identity block can extend further.

Third, further confidence in the *fis attB* comes from previous experimental work (27) showing that the *fis* PICI Kpn47.16.fis in *Klebsiella pneumoniae* BAA-2146 was excised with subsequent replication upon treatment with mitomycin C; excision was shown to have occurred within the identity block TACGGCATGAACTGATAC confirming precisely the site mapped bioinformatically here.

The recognizability of *fis attL* and *attR* sequences makes it unlikely that any elongated *fis* PICI is an unresolved standard PICI/prophage tandem at *fis*, although we did identify and resolve two cases of *fis* PICI/PICI tandems. A remaining possibility for the elongated *fis* PICIs is that a proper PICI/prophage tandem suffered a deletion event across the intervening *att* site, making it challenging to identify the PICI/prophage fusion junction. This fusion hypothesis would require the existence of prophages that can recognize the *fis* site like the PICIs do, which would be proven if we could identify a *fis* gene occupied by a prophage alone. Given the general mosaicism observed among GIs, it is possible that some prophages may have acquired the integration module of these PICIs, but it is also possible that this never occurred in the case of the *fis* integrase. To search for *fis*-integrated prophages, we examined *fis* GIs from recent TIGER results we generated from all 165 778 available proteobacterial genomes. Where our original search was biased by integrase match to that of 11capE, this newer search was unbiased. A total of 3565 *fis* GIs were detected including all 2642 of the original *fis* PICIs. The 923 new *fis* GIs from this Phase 2 survey (Supplementary File 1) mostly had lower bit scores to the 11capE integrase (Supplementary Figure 3C), but others came from the 230 *fis* jobs that had been canceled in the original search; they showed the same *att* site conservation as the Phase 1 *fis* PICIs and also broadened phylogenetic reach beyond the Enterobacteriaceae family, although occurrence was still limited to the Enterobacterales order.

To examine whether any of these new *fis* GIs might be isolated prophages, we used our best signature proteins for distinguishing PICIs from prophages (Table 1). We found that the Pfam Phage_AlpA (the regulatory protein encoded in the PICI Integration zone) hit 462 of the 476 original model PICIs but only one (Ana29.58.R, at a site far from its *int* gene) of the 169 model prophages. Conversely, both of the Pfams Phage_min_tail and Phage_tail_L hit 154 of the model prophages but none of the model satellites. These Pfams were applied to all the new *fis* GI proteins, following up with more sensitive TBLASTN searches because we noticed that some AlpA proteins had been missed by Pfam due to omission of AlpA reading frames during PROKKA gene calling. This search found AlpA matches for six additional satellite models and for all but six of the new *fis* GIs. Five (Hal26.3.fis, Hal10.3.fis, Hal39.3.fis, Sne13.2.fis, Eba181.4.fis) of these *alpA*-lacking GIs were <4 kb and the sixth (Kpn1666.10.fis) had 75.9% of its sequence as ambiguous bases. Phage_tail_L and Phage_min_tail protein yields were unchanged with TBLASTN, and none of the new *fis* GIs were hit. We conclude that prophages (at least of the type that might explain the elongated PICIs) do not use the *fis* site.

**Table 1.** Signature protein gene occurrence. GIs were tested for PROKKA protein matches to one of three Pfams, or for more sensitive TBLASTN match to the protein sets identified by Pfam match. Phage_AlpA proteins are good signatures for the satellites, while Phage_min_tail and Phage_tail_L are good (and equivalent) signatures for the prophages. This identifies the new *fis* GIs as PICIs, not prophages

		GIs with Pfam hits			GIs with TBLASTN hits		
GI group	GIs Tested	Phage_ AlpA	Phage_ min_tail	Phage_ tail_L	Phage_ AlpA	Phage_ min_tail	Phage_ tail_L
Prophage models	169	1	154	154	1	154	154
Original *fis* models	203	203	0	0	203	0	0
All new *fis* GIs	923	888	0	0	917	0	0
Other satellite models	273	259	0	0	265	0	0

A final possibility for the PICI/prophage fusion hypothesis is that the prophage was originally further from the PICI, not adjacent, such that the hypothetical deletion removed intervening genes that are normally downstream of *fis*. For the nine elongated *fis* PICIs, eight were from *Salmonella* strains. From 57705 examined *Salmonella* genomes, 57620 had *yhdJ* as the gene downstream from *fis*. For all eight *Salmonella* elongated PICIs, the gene across the PICI from *fis* was likewise *yhdJ*, indicating no deletion of material that is normally downstream of *fis*. The remaining elongated *fis* PICI (Cnf1.45.fis), from a *Cedecea neteri_C* strain, was followed across from *fis* by the gene sequence HYP/*aapJ/glnM/yhdY/glnQ* (where HYP is a hypothetical gene). The only other available *Cedecea neteri_C* strain (assembly 000758305) had *fis* apparently uninterrupted, followed directly by HYP/HYP/*aapJ/glnM/yhdY/glnQ*, again indicating no significant deletion adjacent to the *fis* PICI.

Eliminating the competing adjacency/fusion hypothesis strengthens the late gene capture hypothesis. Returning to the HE-PICIs and *hpt* PICIs, we note similar confidence in the original calls based on uniformity of *att* identity blocks (Supplementary Files 3 and 4).

## DISCUSSION

We describe a new satellite/helper relationship where the satellite genome lies embedded within its helper prophage genome. Analysis of induction indicates a phase, after excision of the 61icd HE-PICI/helper composite from its integration site in *icd*, when the entire excised composite replicates. To the extent that the helper 50icd component drives this replication, the HE-PICI 11capE is passively replicated as a segment within the larger composite, an example of *cis-*regulation not possible in standard satellite/helper pairs. However 11capE has its own primase and origin of replication, so may well also contribute to replication of itself and the composite.

Additional interactions between the two components of 61icd can be proposed. Certainly the 50icd capsid gene cannot be expressed until the gene is restored by excision of 11capE. This likely reduces 50icd phage yields, in line with other model satellites (reviewed in (11)) known to partially inhibit their helpers. We have discussed how 50icd serves as a helper for 11capE DNA replication, and suspect that 50icd is also the helper for three other functions: (i) detecting the SOS response signals for induction through its cI repressor, (ii) providing the structural proteins and packaging machinery to make 11capE virions, in line with other lambdoid prophage helpers of PICIs and (iii) activating 11capE gene expression in the helper late phase. All of the non-hypothetical 11capE genes are unidirectional, in the same direction as the 50icd capsid gene and its surrounding late genes. It can be hypothesized that late transcription of 50icd will continue into integrated 11capE, transcribing its integrase, capsid and other genes, and will do so at a time when much replication has already occurred. It should be noted that another prophage in these cells, 41torS, is induced earlier, in a larger fraction of the cells, and with faster replication, which may modulate the 61icd activities observed here.

Further analysis was hampered by an inability to detect the expected virions of 50icd, even on lawns of its source strain cured of 61icd, though there may be future paths forward. An interesting remaining question is whether the entire 61icd composite may be packaged into some virions, delivering helper and satellite together into a new host, although this would exceed the insert size limit (∼10% of its genome) that can be tolerated by phage lambda packaging (42). Otherwise, co-delivery to a new host can occur the usual two-virion way, with helper phage virions released together with satellite virions upon lysis.

Surprisingly, a search based on integrase similarity revealed many additional HE-PICIs integrated into diverse prophage late gene sites. This constitutes evidence for a tropism of an integrase clade toward integration sites in prophage late genes. It will be of interest to learn whether mechanistic peculiarities of this integrase subclade account for this tropism. It is possible that the neighboring *alpA* gene, encoding a tightly conserved excisionase/transcription factor, contributes to the tropism. Change at the *attP* core region to accommodate new site specificities could be constrained by the fixed spacing to the surrounding copies of the newly-discovered doubled DNA motif (Supplementary Figure 4), which is unique to the HE-PICIs. Although we found HE-PICIs at 14 *att* sites in nine late genes, 97% of them were at five sites in the four genes A, B, C and E in a core region occupying only 6.5 kb of phage lambda. The most frequent site was in the capsid gene E. This result may be biased, because our search was launched with one of those satellites, yet it has additional significance; capsid genes are the most common late phage gene in GN-PICIs.

We find small numbers of elongated PICIs that have captured large helper phage late gene clusters (Supplementary Figure 6). Continuing adaptation of PICIs to helpers may involve cycles that start with large captures, followed by loss of many of the new genes, but adaptation of some new genes for helper interaction. Late gene capture may be facilitated in HE-PICIs by their localization and recombination activity in these same prophage regions; late genes more likely to be captured are those closest to the HE-PICI integration site. This provides a backup intact copy that may functionally replace the interrupted prophage gene even before HE-PICI excision. This redundancy is not essential; knockout of a capsid gene in a PICI was tolerated (6). Other phage genes can be captured in the Late zone; the integrase phylogenetic tree (Figure 4) shows that the occasional extra integrase genes found in the PICI Late zone are related to those in helper prophages, not to the main PICI integrases. The prophage/satellite gene exchange observed here contrasts with the situation in *Streptococcus*, where prophage/satellite pairs are widespread, but with little genetic exchange (43). The HE-PICI champion is *Arsenophonus nasoniae* FIN, whose genome has four helper prophages each bearing a slightly different B:Phage_portal_2:241 HE-PICI, with one of these helpers additionally bearing a second HE-PICI at a different late gene (Figure 5C).

When an integrase targets a site within a tRNA or protein gene, the *attP* usually carries within itself a replacement sequence from the target gene, positioned such that the post-integration recombinant target gene is still functional. There are exceptions to this rule, that can usually be understood as cases of regulated gene integrity, where disruption of the target gene upon island integration serves to regulate cellular processes such as sporulation and DNA replication fidelity (16,44). HE-PICIs disrupt prophage late genes, but do not carry a replacement sequence in *attP*. Thus HE-PICIs appear to cause regulated gene integrity, in which the regulation serves the HE-PICI, rather than the cell or the prophage. HE-PICIs serve to remind that integrative elements can target not only chromosomal genes but other mobile elements. Interestingly, PICI EcCIDi14 is in a IS110 transposase gene (6).

11capE is presumed to be packaged by the *cos* machinery of the helper, as has been presumed for EcCICFT073 (7), which likewise lacks *terS*; a 258-bp sequence is shared with 90% identity between 11capE and 50icd, at the same site in 11capE where *cos* sites have been identified in other PICIs, and at the location in 50icd that maps to the lambda *cos* site.

Here we show evidence that the phenomenon of damage to the tRNA *attR* gene fragment damage upon integration (35) may be restricted to PICIs, and is not typically exerted by prophages. This damage is possibly adaptive, debilitating the tRNA gene when the PICI excises (17); thus these PICIs can act like an addiction module.

Discovery of GIs, including satellites as well as prophages, is a necessary capability to fully characterize the bacterial genomic landscape. Computational tools for detecting integrated satellites have been lacking (45), but this work shows that TIGER/Islander software is suitable for fully automated and precise mapping, in spite of their small sizes and ability to use rare integration sites. Scale-up of our software to all available bacterial genomes is expected to identify numerous satellites in other bacterial groups.

PICIs and HE-PICIs exemplify different integration strategies. PICIs typically target conserved housekeeping genes, where they may wait for arrival of the helper if it is not already there. In contrast for HE-PICIs, when the target site is available (helper is present) it favors an especially profitable HE-PICI location that can provide several *cis-*regulatory advantages. However, the target site will be more often absent for a HE-PICI than for a PICI, because the HE-PICI helper may be absent or its target late gene may be more subject to change than a housekeeping gene. This may explain our finding of several off-target HE-PICI integration events. Interestingly, no off-target relatives were found for the 2701 PICIs using the chromosomal sites *fis* or *hpt*.

Inspection of annotations showed that many of the HE-PICI helpers appeared truncated in their chromosomal setting; such potentially abandoned HE-PICIs may retain functions allowing activation when a new helper enters the cell. The local source of phage genes in the helper may facilitate the recombinational shuffling of phage genes into HE-PICIs. The HE-PICIs, PICIs, P4 and the *Vibrio* PLEs are rich in Bro network proteins and will provide an excellent system to better characterize their interesting domain-swapping mechanisms and evolutionary history.

HE-PICIs are moderately widespread. We estimate that among the *E. coli* genomes, where chances were best for finding 11capE-related HE-PICIs, ∼5.4% of genomes contain a HE-PICI (this calculation accounted for the high fraction, 56%, of 11capE *int* hits that fell in a region unsuitable for HE-PICI detection due to contig truncation or ambiguous bases).

## DATA AVAILABILITY

Sequencing read sets have been deposited in the Sequence Read Archive (PRJNA928767).

## Supplementary Material

lqad036_Supplemental_Files
