# Supplementary material for: Helper-embedded satellites from an integrase clade that repeatedly targets prophage late genes
Source: NAR Genom Bioinform. 2023 Apr 18;5(2):lqad036. doi: 10.1093/nargab/lqad036 (PMC10111431; doi:10.1093/nargab/lqad036)

Supplementary materials for "Helper-embedded satellites from an integrase clade that repeatedly targets prophage late genes",

Dario Tommasini, Catherine M. Mageeney and Kelly P. Williams, 2023.

SUPPLEMENTARY FILES

Supplementary File 1 (separate spreadsheet file). Genomic island study set. Sheet "Phase 1 GIs": 3527 genomic islands (GIs), discovered based on integrase similarity to that of 11capE or from previous references. Accession:coordinates, attachment site identity sequences and other information. All are from the family Enterobacteriaceae except for some reference PICIs from the Pasteurellaceae. Sheet "Phase2 New *fis* GIs": 923 additional GIs integrated into the *fis* gene, discovered without bias by TIGER from search of 165,778 Proteobacteria genomes. Accession:coordinates, attachment site identity sequences and other information. Some reach outside the Enterobacteriaceae. Sheet "Gene Profiles": 645 unique gene profiles for the fully-mapped Phase 1 GIs. Those for the prophage helpers of HE-PICIs are for the hypothetical form with the HE-PICI deleted.

Supplementary File 2 (separate protein sequence alignment file). Bro network protein segments.

Supplementary Files 3 and 4 (separate nucleotide sequence alignment files). Attachment sites. The *attL* (File 1) and *attR* (File 2) sequences of each model GI are shown, not fully aligned but mainly shifted to optimize alignment of the identity block (upper case) within in each group. The 14 HE-PICI groups with their off-site relatives are at the top, followed by PICI groups, then prophage groups. All tRNA sites used are at the bottom, with the complete tRNA sequence in *attL* aligned based on secondary structure, and the *attR*s aligned to emphasize damage (small deletions beginning at position 99) at the 3′ portions of the Pro, Thr, SeC and some Ser and Ile2 tRNAs [Williams KP. Integration sites for genetic elements in prokaryotic tRNA and tmRNA genes: sublocation preference of integrase subfamilies. Nucleic Acids Res 2002;30:866-875].

SUPPLEMENTARY TABLES

Supplementary Table 1. Phase 1 bioinformatic identification of HE-PICIs and other GIs.

	Founder *int* Regions	HE-PICI/ tRNA-Phe PICI	fis/hpt/ tRNA-Met PICI	HE-PICI-Surrounding Prophage	HE-PICI-Surrounding PICI	Total GIs Discovered
Discover founder HE-PICI 11capE and its helper 61icd		1		1		2
Use 11capE integrase as TBLASTN query vs. 165778 proteobacterial genomes with cutoff of 400 bits	5068	1		1		2
Select those with intact ~22-kbp regions	3490	1		1		2
Early TIGER runs reveal many regular *fis* PICIs; cancel 230 remaining *fis* region jobs	3260	1		1		2
TIGER yields on regions	3260	505	2672	1		3178
Run TIGER on whole genome	3260	519	2690	1		3210
Manual inspection allows full attL/R mapping	3260	527	2690	1		3218
Manual inspection allows attL-only mapping	3260	538	2713	1		3252
Integrase tree assigns family to 9 unmapped	3260	545	2715	1		3261
Split three tandems manually	3260	545	2718	1		3264
TIGER/Islander whole-genome search for HE-PICI-surrounding GIs	3260	545	2718	208	24	3495

Supplementary Table 2. Primers used in this study

**Oligonucleotide**	**Sequence**	**Use**
143.11capE.CJ.L	cggccacctgctaacctgta	Identifying 11capE attL, attP
144.11capE.CJ.R	agttttgatgctcgtttgatatccc	Identifying 11capE attR, attP
145.50icd.CJ.L	gcgatctctgtcagaacggt	Identifying 50icd attL, attP; qPCR
146.50icd.CJ.R	gagacacataaggcctcgca	Identifying 50icd attR, attP
147.Eco567.icd.DJ.L	acccgaataccggcaaagag	Identifying 50icd attL, attB
148.Eco567.icd.DJ.R	aagaggccgcattgcttcat	Identifying 50icd attR, attB
149.Eco567.capE.DJ.L	gccggaacggcaatcagc	Identifying 11capE attL, attB
150.Eco567.capE.DJ.R	cagcgcgtaggcttcgatatcg	Identifying 11capE attR, attB
319.11capE_Q_CJ_L	aggaaaattcagtgaaagcgcc	Quantifying 11capE attL, attP
320.11capE_Q_CJ_R	tggttgcatcgtgtcatcct	Quantifying 11capE attR, attP
321.11capE_Q_DJ_L	aacatggcgctgtacgtttc	Quantifying 11capE attL, attB
322.11capE_Q_DJ_R	gcctgcatctcttcgacctg	Quantifying 11capE attR, attB
355.Eco567.icd_DJ_L_qPCR	atgccggacaggacaaagta	Quantifying 50icd attL, attB
356.50icd_CJ_R_qPCR	cgaacgtcaatgaaatcaaacggt	Quantifying 50icd attR, attP
357.Eco567.icd_DJ_R_qPCR	gctccccataaataatcaccagac	Quantifying 50icd attR, attB
366.Eco567_Q_polymerase_L	taacctggtcgggctttctt	Quantifying DNA polymerase I
367.Eco567_Q_polymerase_R	gcttcttcgagggcaatctg	Quantifying DNA polymerase I

Supplementary Table 3. Bioinformatic probes used with AttCt to detect predicted GIs and housekeeping gene *polA* (DNA polymerase I).

**Target**	**attB**	**attP**	**attL**	**attR**
polA	N/A	N/A	taacctggtcgggc	gcttcttcgagggc
61icd	gatgctgcgccaca	actgctgcgccata	gatgctgcgccata	actgctgcgccaca
11capE	aaacccaagcatg	catgtaacccaagcacttag	aaacccaagcactt	taacccaagcatga
41torS	gcactttaggtgaaaaaggttgagt	atcctttaggtgaataagttgtata	gcactttaggtgaataagttgtata	atcctttaggtgaaaaaggttgagtc
45proP	gcttgaattatggacttccagttat	ctgtgaatttatggatttcaagagt	gcttgaatttatggatttcaagagt	ctgtgaattatggacttccagttat
2emrE	caccacattaaaaataatttatttttaaacgactaaaataggtt	gcccccattaaaaataatttatttttaaacgactaaaatatgga	caccacattaaaaataatttatttttaaacgactaaaatatgga	gcccccattaaaaataatttcttttgaatcgagtgaaataggtt
48F	caggggattgaaaatccccgtgtccttggttcgattccgagtccgggcaccaaat	tcagggattgaaaatccccgtgtccttggttcgattccgagtccgggcaccacta	caggggattgaaaatccccgtgtccttggttcgattccgagtccgggcaccacta	tcagggattgaaaatccccgtgtccttggttcgattccgagtccgggcacccaaat
7betA	aatactggatatgaagcatga	gattgtcacgatttatagcct	aatactggatatttatagcct	gattgtcacgatgaagcatga
4fimD	atgcagggcgtttacgcgaca	gtgcgtggcgtttttacggca	atgcagggcgtttttacggca	gtgcgtggcgtttacgcgaca

SUPPLEMENTARY FIGURES

Supplementary Figure 1. Late/early genes in co-oriented clusters. The 168 gene profiles for prophages were split into uninterrupted clusters of co-oriented genes, collecting those containing a holin/tail seed family as "late clusters". Based on transcription profiles for reference phages lambda and P2, non-seed families were labeled "Known Early" or "Known Late" and the remainder "Test".

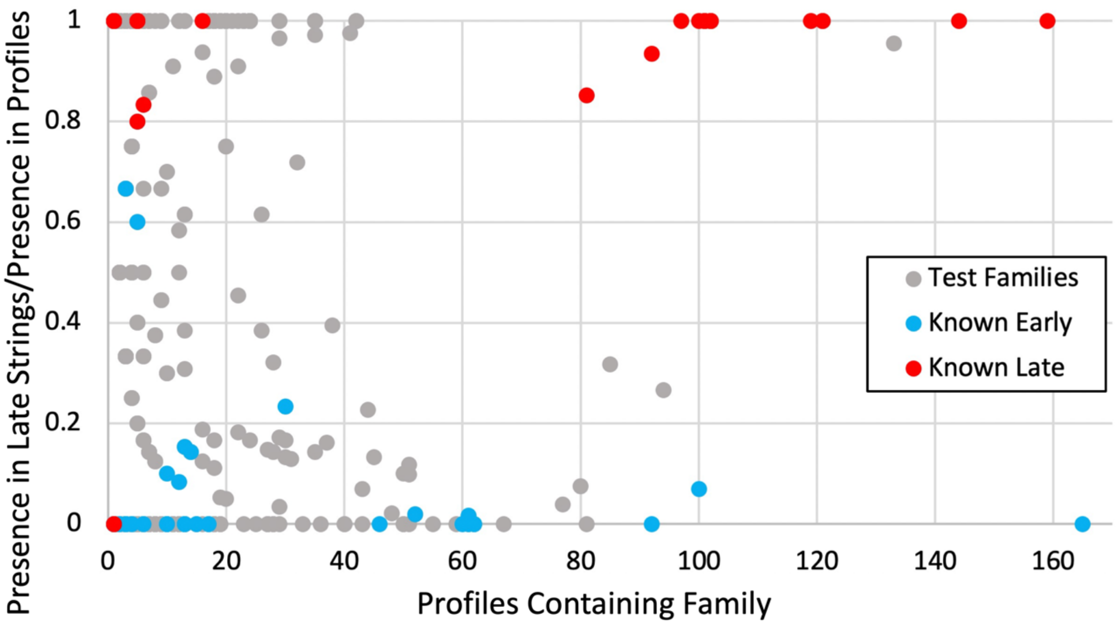

Supplementary Figure 2. PCR products used as standards in qPCR experiments. Agarose gel showing PCR products for the eight attachment sites of interest and the distant *polA* housekeeping gene. These products were used as standard templates after dilution to $1x10^{-2}$ ng, $1x10^{-4}$ ng, $1x10^{-6}$ ng, and $1x10^{-8}$ ng. They were amplified and standard curves were generated by plotting Ct value with respect to the logarithm of the initial mass.

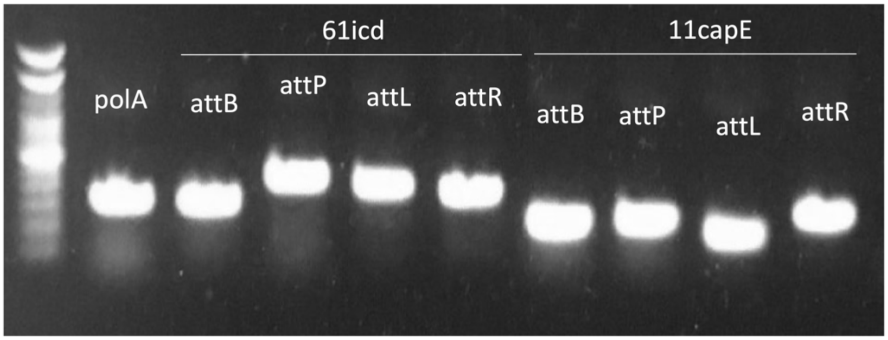

Supplementary Figure 3. Integrases related to that of 11capE. A) Databases containing a total of 165,778 proteobacterial genomes were queried with the 11capE integrase sequence using TBLASTN, binning hits by bit score. Each of the 5068 integrase genes above (green) a 400-bit cutoff (dashed line) was used to initiate the Phase I search for a genomic island; those below cutoff (red) were excluded from Phase 1. B) *fis* GIs either found originally in regions with integrases above cutoff (orange) or new ones found in whole genome searches of all proteobacterial genomes (blue) were treated as above, only recording the top hit for the GI (because some have other secondary integrase genes). The two orange points below cutoff are from the second members of the two *fis* tandems. C) Sequence conservation at the *fis att* site. The 12 unique slight variants of the 15-mer observed at the *attL* and *attR* sites found by TIGER were equally weighted. Sequence is shown in the sense of the *fis* gene (PICIs have the opposite orientation).

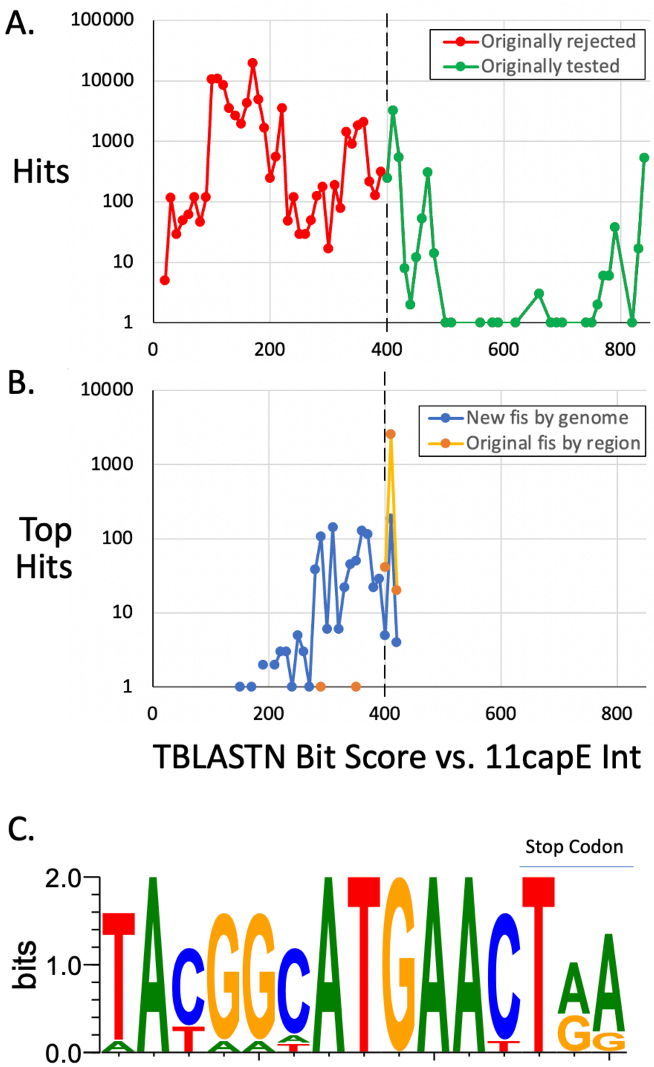

Supplementary Figure 4. Doubled DNA sequence motif found at two sites in each HE-PICI *attP*. Sequence logos are shown for the two double-tgTGTATTGCa motifs found at remarkably well-defined distances from the *attP* center. Orientation is that of the integrase and most other HE-PICI genes. The motif was not found in other GI types besides HE-PICIs.

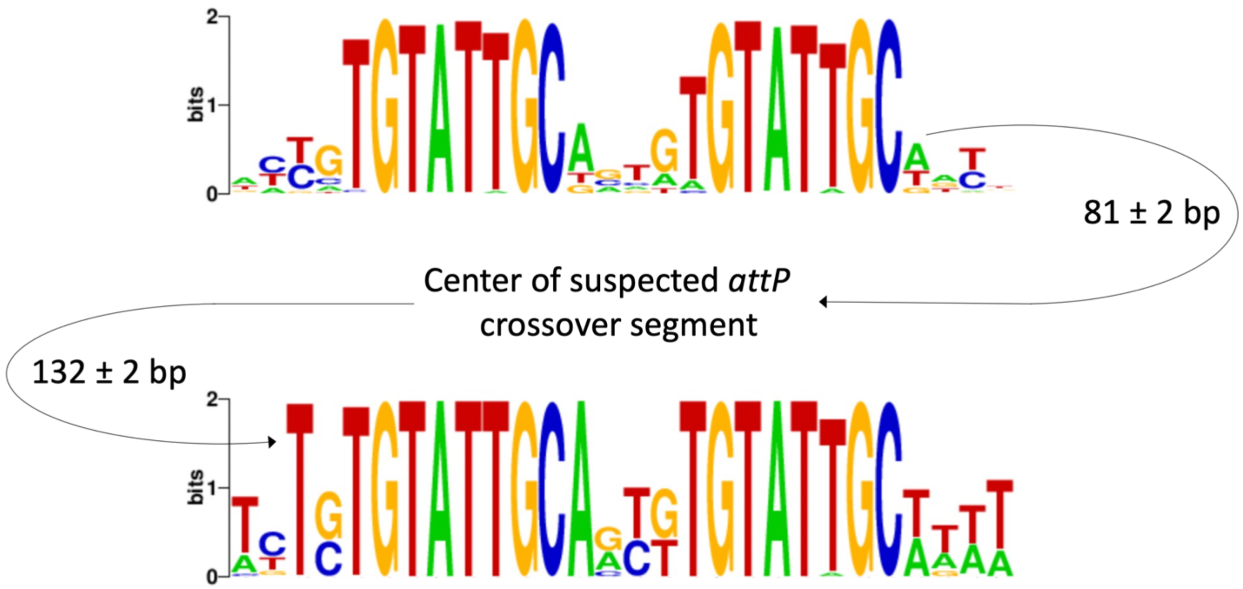

Supplementary Figure 5. Gene maps for model GIs. **A**) 220 HE-PICIs and close relatives (off-targets and tRNA-Phe PICIs). **B**) 256 other satellites (P4, fis/hpt PICIs, and reference gram-negative PICIs). **C**) 169 HE-PICI helper prophages (site of HE-PICI marked by downward black arrow), as well as lambda, P2 (helper of P4) and Eco150.55.icd (helper of GN-PICI EcCICFT073); some apparent gene-empty prophage regions are due to long gaps (N-blocks) in the genome sequences. Purple diamonds mark the 11 PICIs longer than 22 kbp, all expanded in the Late zone. Accession:coordinates and other information in Supplementary File 1.

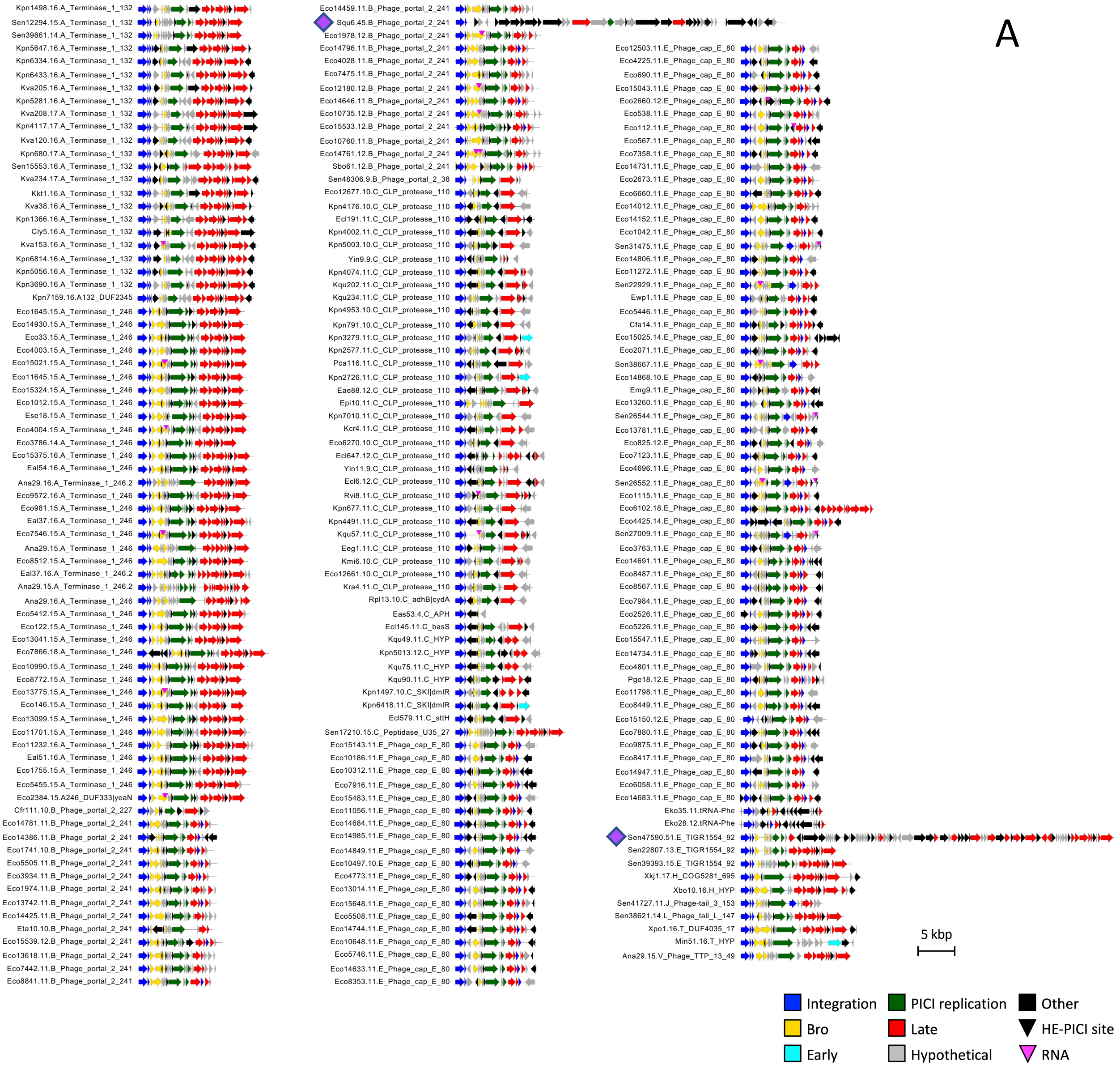

Supplementary Figure 5, panel B (other satellites).

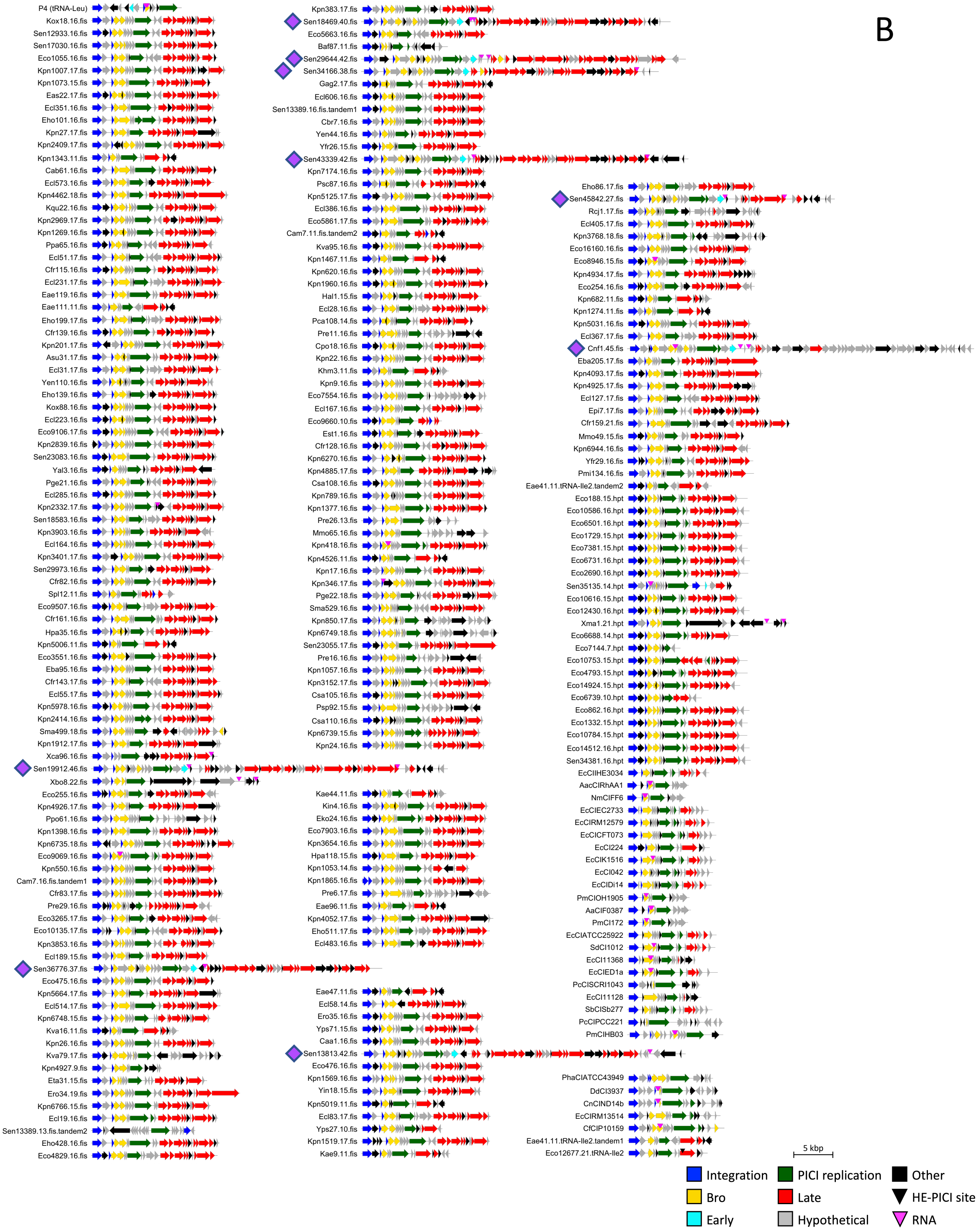

Supplementary Figure 5, panel C (helper prophages).

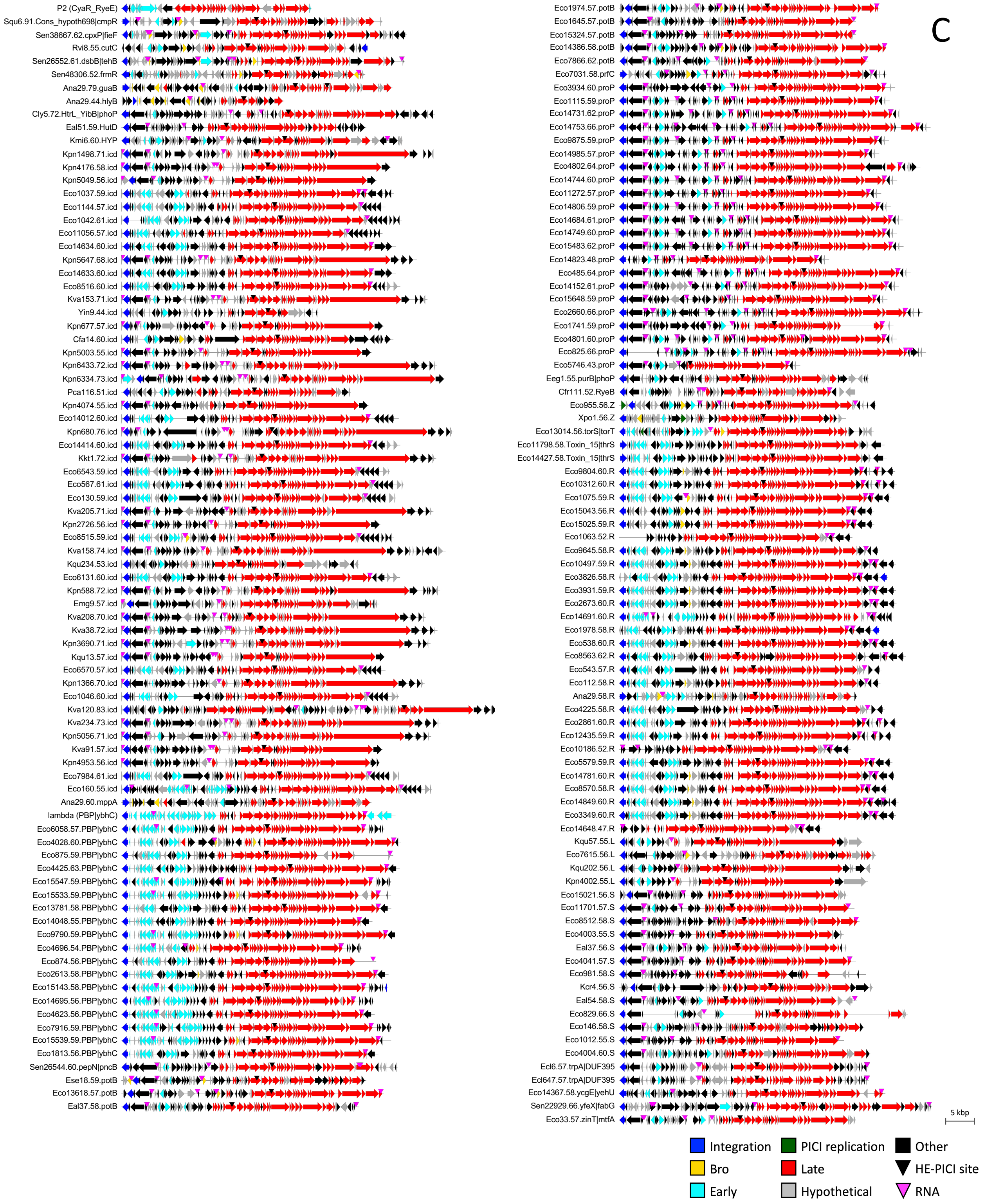

Supplementary Figure 6. Late gene capture. Six PICIs modeling late gene capture are shown. The seven shared gene clusters of five or more same-annotated genes (blue boxes, noting the matching prophages) were all from late zones of elongated PICIs. Stronger PICI/prophage matches were revealed by BLASTN (pink parallelograms, percent identities in parentheses). In the two cases where the BLASTN match touches an HE-PICI integration site within a prophage, the map of that prophage is also shown.

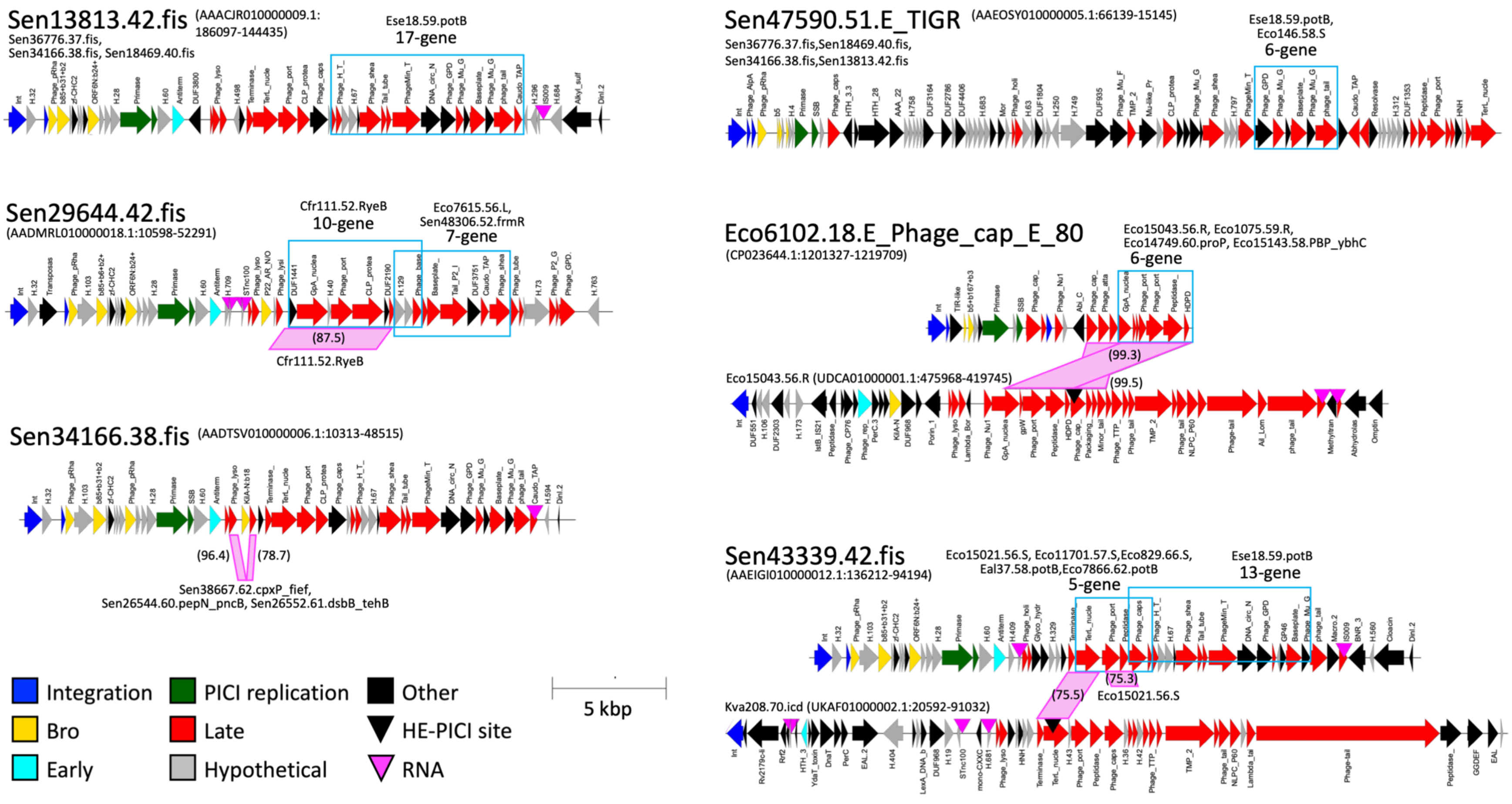

Supplement: lqad036_Supplemental_Files [file lqad036_supplemental_files.zip › Supp.pdf]
